# Glomerular deposition of galactose-deficient IgA1-containing immune complexes via glomerular endothelial cell injuries

**DOI:** 10.1093/ndt/gfac204

**Published:** 2022-06-23

**Authors:** Yuko Makita, Hitoshi Suzuki, Daisuke Nakano, Hiroyuki Yanagawa, Toshiki Kano, Jan Novak, Akira Nishiyama, Yusuke Suzuki

**Affiliations:** Department of Nephrology, Juntendo University Faculty of Medicine, Tokyo, Japan; Department of Nephrology, Juntendo University Faculty of Medicine, Tokyo, Japan; Department of Nephrology, Juntendo University Urayasu Hospital, Chiba, Japan; Department of Pharmacology, Kagawa University, Kagawa, Japan; Department of Nephrology, Juntendo University Faculty of Medicine, Tokyo, Japan; Department of Nephrology, Juntendo University Faculty of Medicine, Tokyo, Japan; University of Alabama at Birmingham, Birmingham, AL, USA; Department of Pharmacology, Kagawa University, Kagawa, Japan; Department of Nephrology, Juntendo University Faculty of Medicine, Tokyo, Japan

**Keywords:** endothelial cell, Gd-IgA1, glycocalyx, IgA nephropathy

## Abstract

**Background:**

Galactose-deficient immunoglobulin A1 (Gd-IgA1) plays a crucial role in the development of IgA nephropathy (IgAN). However, the pathological role of Gd-IgA1-containing immune complexes (ICs) and the mechanism of deposition in the mesangial region remain unclear.

**Methods:**

To examine the deposition of Gd-IgA1-containing ICs in the mesangial region through glomerular endothelial cell injury, we evaluated the alteration of renal microvascular endothelial glycocalyx in nude mice injected with Gd-IgA1-IgG ICs. Human renal glomerular endothelial cells (HRGECs) were used to assess the potential capacity of Gd-IgA1-IgG ICs to activate endothelial cells.

**Results:**

Nude mice injected with Gd-IgA1-containing ICs showed podocyte and endothelial cell injuries, with IgA, IgG and C3 depositions in glomerular capillaries and the mesangium. Moreover, albuminuria and hematuria were induced. Real-time glycocalyx imaging showed that renal microvascular glycocalyx was decreased immediately after injection of Gd-IgA1-containing ICs and then mesangial IgA deposition was increased. After coculture of Gd-IgA1-containing ICs with HRGECs, messenger RNA expression levels of endothelial adhesion molecules and proinflammatory mediators were upregulated significantly.

**Conclusion:**

Gd-IgA1-IgG ICs had a high affinity for glomerular endothelial cells, which resulted in glomerular filtration barrier dysfunction mediated by glycocalyx loss. Furthermore, Gd-IgA1-IgG ICs accelerated the production of adhesion factors and proinflammatory cytokines in glomerular endothelial cells. The glomerular endothelial cell injury induced by Gd-IgA1-containing ICs may enhance the permeability of Igs in the mesangial region and subsequent inflammatory responses in the pathogenesis of IgAN.

## Graphical Abstract

**Graphical Abstract:**
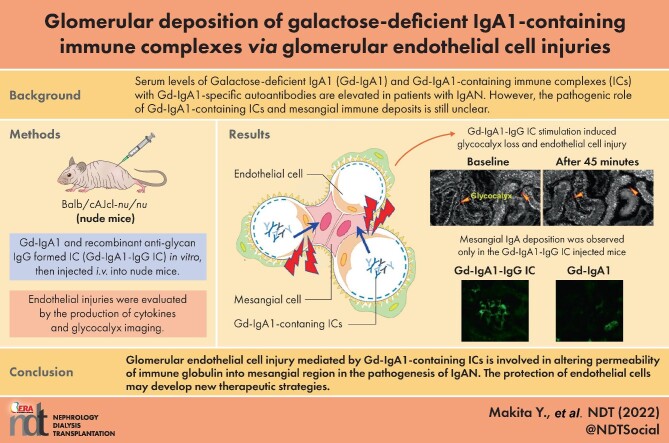

KEY LEARNING POINTS **What is already known about this subject?**

- Immunoglobulin A (IgA) nephropathy (IgAN) shows galactose-deficient (Gd)-IgA1 deposition, and notably, Gd-IgA1 plays a crucial role in IgAN development.
- However, the mechanism of Gd-IgA1 deposition in the mesangial region is uncertain.

**What this study adds?**

- This study shows that Gd-IgA1-containing immune complexes (ICs) have an affinity for glomerular endothelial cells and induce endothelial cell injury.
- Gd-IgA1-containing ICs induce the production of inflammatory cytokines and adhesion molecules in endothelial cells and result in glomerular filtration barrier dysfunction mediated by glycocalyx loss.

**What impact this may have on practice or policy?**

- These results provide a new therapeutic strategy that targets maintenance of endothelial cell functions to prevent deposition of Gd-IgA1 in the mesangial region in the pathogenesis of IgAN.

## INTRODUCTION

Immunoglobulin A nephropathy (IgAN) is a common primary glomerulonephritis that can lead to kidney failure in 20–40% of patients within 20 years after onset [1, 2]. IgAN is defined by the presence of mesangial IgA-containing immunodeposits, usually with complement 3 (C3). Glomerular IgA in IgAN patients is restricted to the IgA1 subclass and is enriched with molecules with some hinge-region galactose-deficient *O*-glycans, so-called galactose-deficient IgA1 (Gd-IgA1) [3, 4]. Prior studies have shown elevated serum levels of Gd-IgA1 and Gd-IgA1-containing immune complexes (ICs) with Gd-IgA1-specific IgG autoantibodies in IgAN patients [5–7]. Furthermore, immunofluorescence analyses of renal biopsy specimens from IgAN patients have shown glomerular deposition of Gd-IgA1 [8]. However, the mechanisms that underlie mesangial deposition of IgA1 and Gd-IgA1 ICs remain unclear.

In a previous study, we demonstrated that nephritogenic IgA and IgA–IgG ICs isolated from IgAN-prone mice and injected into nude mice resulted in rapid glomerular deposition of IgA along the glomerular capillary wall and in the mesangium [9]. These findings indicated that nephritogenic IgA ICs may interact with glomerular endothelial cells prior to depositing in the mesangial region. Furthermore, we have shown that *in vitro*–formed human Gd-IgA1-IgG ICs injected into nude mice formed glomerular immunodeposits and induced pathologic changes typical for human IgAN [10].

Glomerular endothelial cells regulate vascular functions, such as controlling the permeability of small solutes, proteins and inflammatory cells entering and leaving the bloodstream [11]. The luminal surface of vascular endothelial cells is covered by endothelial glycocalyx that provides a barrier against free passage of proteins and prevents the adherence of inflammatory cells to the endothelium [12]. Glycocalyx injury would allow proteins to pass through the endothelial fenestration and enable transmigration of inflammatory cells through the endothelium [13, 14]. Endothelial glycocalyx has been suggested to be a major determinant of vascular dysfunction manifesting with hyperpermeability in albuminuric kidney disease [15]. Therefore, evaluation of endothelial glycocalyx loss may provide insights into increased glomerular permeability.

Mesangial deposits of IgA-containing ICs are thought to originate from the circulation, but details and mechanisms are not well understood in IgAN. We hypothesize that mesangial deposition of Gd-IgA1-containing ICs is mediated by glomerular endothelial cell injury. To test the hypothesis and gain a better understanding of the underlying mechanisms involved in endothelial cell injury, we used a recently published model wherein human Gd-IgA1-IgG ICs are injected into nude mice to form glomerular immunodeposits and induce pathologic changes resembling those in human IgAN [10].

## MATERIALS AND METHODS

### Animals and experimental protocols

Twelve-week-old athymic Balb/cAJcl-nu/nu (nude) female mice (SLC Japan, Shizuoka, Japan) were used because of their reduced ability to form antibodies against heterologous proteins. Nude mice were maintained at the animal facility of Juntendo University, Tokyo, Japan. The experimental protocol was approved by the Ethics Review Committee for Animal Experimentation of Juntendo University. We used a recently published model, with modifications, wherein human Gd-IgA1-IgG ICs are injected into nude mice to form glomerular immunodeposits and induce pathologic changes resembling those in human IgAN [10]. ICs were formed *in vitro* by overnight incubation at 4°C of purified polymeric Gd-IgA1 myeloma protein (Ale) with purified serum IgG from patients with IgAN that contained Gd-IgA1-specific autoantibodies or IgG from sera of healthy controls, as reported earlier [10]. Polymeric IgA1 (Ale) myeloma protein is naturally galactose-deficient in some *O*-glycans and without sialic acid on most of these *O*-glycans [10]. Notably, this polymeric Gd-IgA1 (Ale) is recognized by IgG autoantibodies extracted from glomerular deposits of kidney biopsy specimens from patients with IgAN [16]. Nude mice were injected once with 250 µg of Gd-IgA1-IgG ICs, Gd-IgA1 + IgG, Gd-IgA1 or normally glycosylated human IgA1 (normal IgA) (Abcam, Cambridge, UK) (five mice for each condition). Real-time glycocalyx imaging was performed using two mice from each group. The mice were sacrificed and their kidneys were harvested 2 hours after administration of ICs or IgA proteins alone to examine mesangial deposition and kidney injuries. Urine samples were collected to assess urinary protein and hematuria.

Kidneys were removed after perfusion with normal saline. Renal specimens for immunofluorescence were immersed in OCT compound (Sakura Finetek Japan, Tokyo, Japan) and stored at −80°C. The specimens were cut into 3-µm-thick sections, fixed with acetone at −20°C for 5 minutes, washed with phosphate-buffered saline, blocked with bovine serum albumin (DS Pharma Biomedical, Osaka, Japan) at room temperature for 30 minutes and then incubated with the following primary antibodies: fluorescein isothiocyanate (FITC)-conjugated polyclonal rabbit anti-human IgA (Dako, Santa Clara, CA, USA), FITC-conjugated polyclonal rabbit anti-human IgG (Jackson Laboratory, West Grove, PA) and FITC-conjugated monoclonal rabbit anti-mouse C3 (Santa Cruz Biotechnology, Dallas, TX, USA). Endothelial cell injury and electron-dense deposits were observed by transmission electron microscopy.

### Real-time glycocalyx imaging

Real-time glycocalyx imaging was performed as described previously [15]. FITC-labeled wheat germ agglutinin lectin (from *Triticum vulgaris* L4895; Sigma-Aldrich, St. Louis, St. Louis, MO, USA) was used to label *N*-acetyl glucosamine moieties of glycocalyx. Nude mice were administrated 250 µg of Gd-IgA1-IgG ICs, Gd-IgA1 + IgG, Gd-IgA1 or normal IgA via the carotid cannula. After observing glycocalyx for 45 minutes, the mice were sacrificed 2 hours later to assess the formation of glomerular immunodeposits.

### Cell culture

Human renal glomerular endothelial cells (HRGECs) (KAC, Tokyo, Japan) were cultured in CS-C Serum-Free Complete Medium (KAC) supplemented with 10% fetal calf serum, 1% streptomycin and 1% penicillin in a humidified incubator at 37°C with 5% carbon dioxide. Cells were cultured after supplementation of Gd-IgA1 alone, Gd-IgA1-IgG ICs or normal IgA for 72 hours.

### Real-time quantitative reverse transcription polymerase chain reaction (RT-PCR)

RNA from HRGECs was extracted using TRIzol (Invitrogen, Tokyo, Japan) and purified with an RNeasy Mini Kit (74106; Qiagen, Valencia, CA, USA). Real-time RT-PCR was performed using the 7500 Real-Time PCR System with SYBR Green PCR Master Mix (Applied Biosystems, Tokyo, Japan). The primers used were as follows: tumor necrosis factor-α (TNF-α) forward primer: TCTCCCAACATGGTTCTCCGTCG, reverse primer: TGCAGTCCAGGCCATGA; interleukin-6 (IL-6) forward primer: GCACCTGTGTCTGGTCCATT, reverse primer: CTGTTGGACACCTGGAGACA; vascular cell adhesion molecule-1 (VCAM-1) forward primer: GCACCTGTGTCTGGTCCATT, reverse primer: CTGTTGGACACCTGGAGACA; intercellular adhesion molecule-1 (ICAM-1) forward primer: GCACCTGTGTCTGGTCCATT, reverse primer: CTGTTGGACACCTGGAGACA; E-selectin forward primer: GCACCTGTGTCTGGTCCATT, reverse primer: CTGTTGGACACCTGGAGACA; and housekeeping gene glyceraldehyde-3-phosphate dehydrogenase forward primer: CATTGTGGAAGGGCTCATGA, reverse primer: TCTTCTGGGTGGCAGTGATG. The amplification conditions were as follows: preheating at 95°C for 20 seconds and 40 cycles of denaturation at 95°C for 3 seconds and annealing and extension at 60°C for 30 seconds.

### Statistical analysis

Correlations between the different parameters were examined by analysis of variance. Data were expressed as the mean ± standard deviation (SD) or median values. *P*-values <.05 were considered statistically significant. All statistical analyses were performed using GraphPad Prism version 9.0 for Windows (GraphPad Software, San Diego, CA, USA). *P*-values were calculated using an unpaired nonparametric >*t*-test (Mann–Whitney).

## RESULTS

### Gd-IgA1-IgG ICs produce glomerular immunodeposits with C3 and induce renal injury

**Figure 1:**
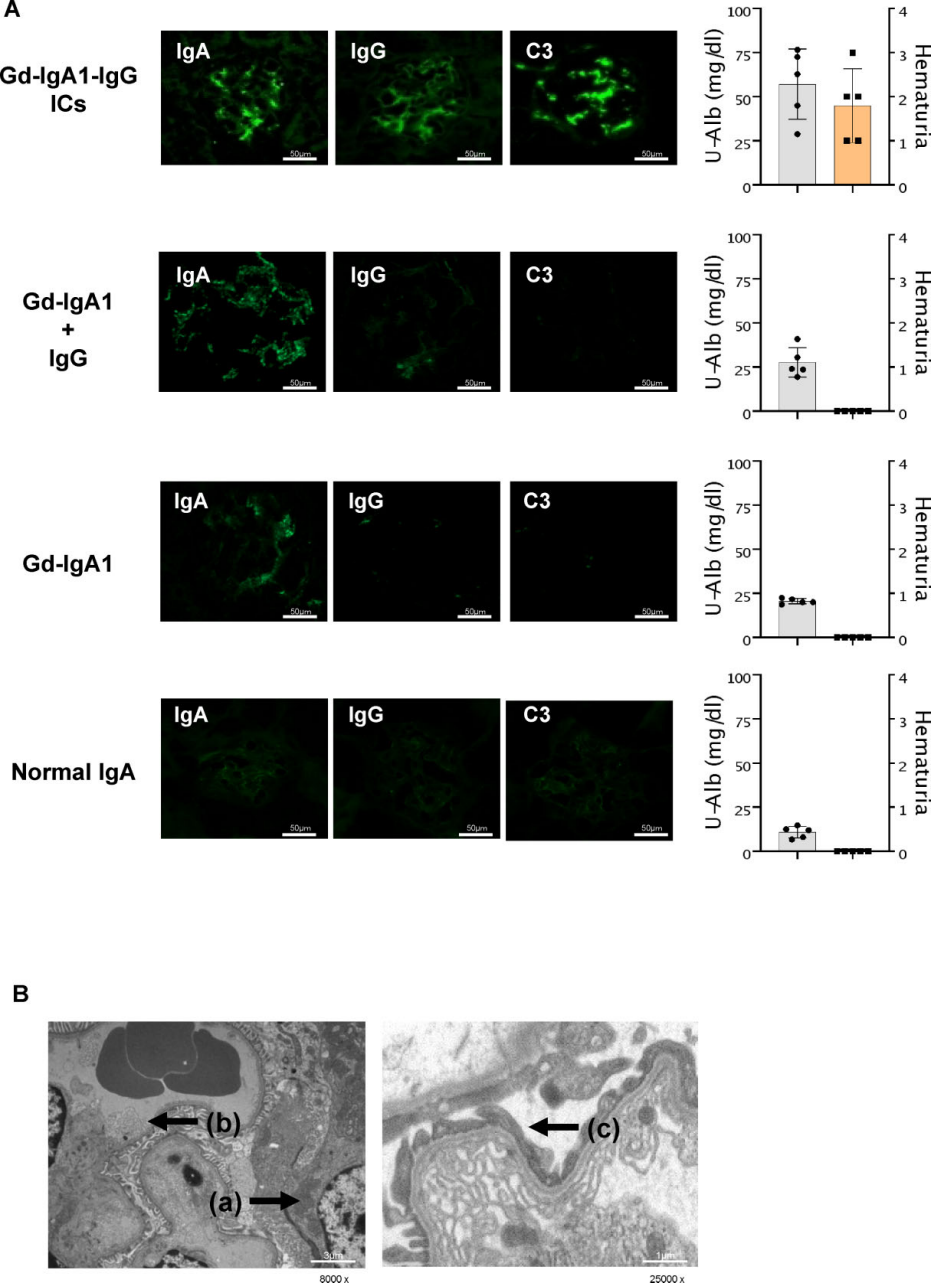
Gd-IgA1-IgG IC stimulation aggravates kidney injuries. **(A)** Nude mice were injected with Gd-IgA1-IgG ICs or control preparations. Immunofluorescence analysis revealed glomerular deposits consisting of IgA, IgG and C3 deposits but only in mice injected with Gd-IgA1-IgG ICs. Urinary albumin and hematuria in Gd-IgA1-IgG IC-injected mice were elevated compared with control mice. **(B)** Electron microscopic examination showed electron-dense deposits localized in the mesangium (arrow (a)). Furthermore, foot process effacement (arrow (b)) and arcade formation (arrow (c)) were observed. These results indicate that Gd-IgA1-IgG ICs induced cell injury in endothelial cells and podocytes. Original magnification, ×8000 or ×25 000 as displayed in the figures. Size bars are provided for each figure in each panel.

Gd-IgA1-IgG ICs were injected into nude mice to examine their capacity to form glomerular immunodeposits and induce renal injury, as we have previously reported [10]. Gd-IgA1-IgG IC-injected mice developed mesangial immunodeposits of IgA, IgG and C3 (Figure 1A) and had elevated albuminuria and hematuria compared with mice that were injected with Gd-IgA1 + IgG, Gd-IgA1 alone or normal IgA (Figure 1A). Electron microscopy revealed electron-dense deposits in the subendothelial area of glomerular capillaries with evidence of podocyte and endothelial cell activation, such as foot process effacement and arcade formation, respectively (Figure 1B).

### Gd-IgA1-IgG ICs induce removal of glycocalyx in renal microvasculature

**Figure 2:**
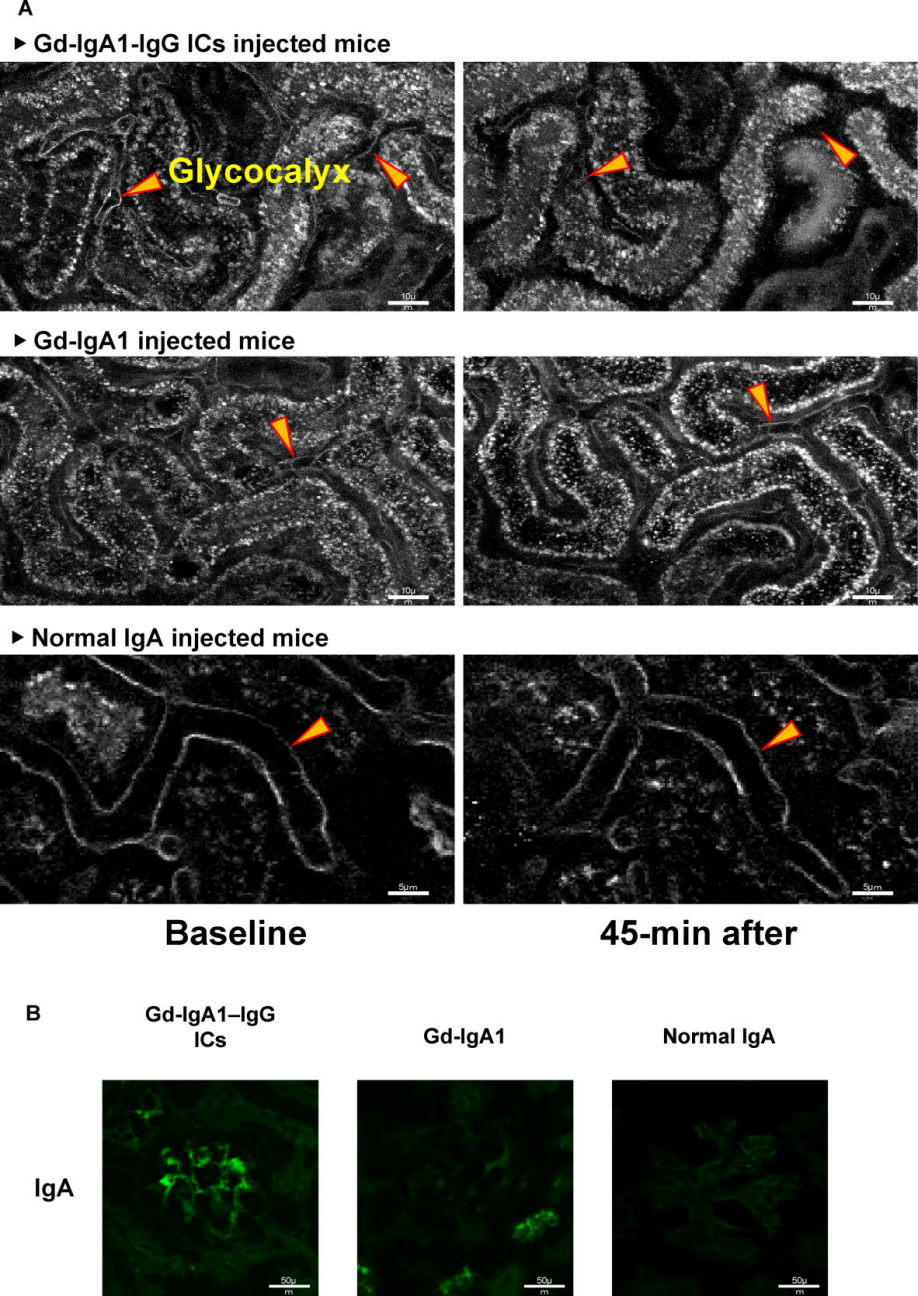
Gd-IgA1-IgG ICs remove renal microvascular glycocalyx. **(A)** Nude mice were injected with Gd-IgA1-IgG ICs, Gd-IgA1 or normal IgA via the carotid cannula. Injection of Gd-IgA1-IgG ICs resulted in rapid glycocalyx removal and the glycocalyx loss was maintained during the 45-minute observation. **(B)** Gd-IgA1-IgG IC-injected mice, but not mice injected with IgA only, developed mesangial deposits of IgA. Size bars are provided for each figure in each panel.

Endothelial glycocalyx that covers the luminal surface of glomerular endothelial cells regulates glomerular permeability [15]. In this study, glycocalyx loss was evaluated by real-time imaging to assess endothelial cell injury that enhances glomerular permeability. Immediate removal of renal microvascular glycocalyx was observed along with the development of concurrent Gd-IgA1-IgG IC deposits in nude mice. Glycocalyx loss was maintained during the 45-minute observation period (Figure 2A). Conversely, mice injected with only Gd-IgA1 did not lose glycocalyx. Furthermore, mesangial deposition of IgA was observed only in Gd-IgA1-IgG IC-injected mice (Figure 2B). These findings suggest that Gd-IgA1-IgG ICs disrupt the barrier of the endothelial surface layer, which may enhance permeability to the mesangial region and subsequent mesangial IgA deposition.

### Gd-IgA1-IgG ICs induce production of inflammatory mediators in cultured human renal glomerular cells

**Figure 3:**
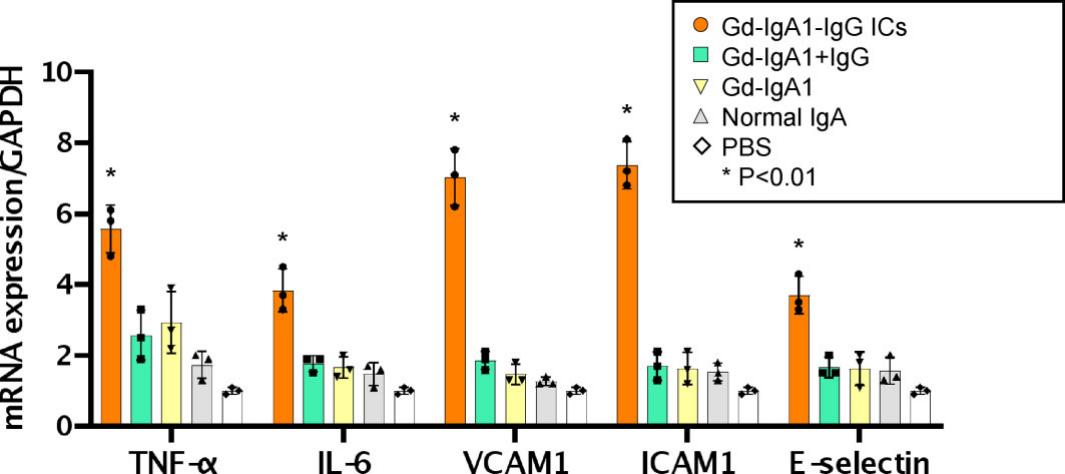
Gd-IgA1-IgG ICs enhance the production of proinflammatory mediators in cultured HRGECs. HRGECs were cultured with Gd-IgA1-IgG ICs, Gd-IgA1 or normal IgA for 72 hours. Gd-IgA1-IgG ICs elevated transcription of endothelial adhesion factors VCAM-1, ICAM-1 and E-selectin in HRGECs. TNF-α and IL-6, proinflammatory mediators that induce adhesion factors expression, were also increased by Gd-IgA1-IgG ICs. Bars represent the mean ± standard error of the mean. *P < .01.

Next we evaluated whether Gd-IgA1-IgG ICs can activate endothelial cells using cultured HRGECs. Gd-IgA1-IgG ICs upregulated the transcription of genes encoding endothelial adhesion molecules, such as VCAM-1, ICAM-1 and E-selectin, in HRGECs (Figure 3). Furthermore, transcription levels of TNF-α and IL-6, proinflammatory mediators that induce the expression of adhesion factors in endothelial cells, were elevated (Figure 3).

## DISCUSSION

IgA1 with aberrant *O*-glycans is characteristic for nephritogenic IgA1-containing ICs [7]. A recent study detected Gd-IgA1-specific IgG in all IgAN patients, indicating that Gd-IgA1 and Gd-IgA1-specific IgG are molecules of significant interest in the process of glomerular deposition [16]. In this study, injection of Gd-IgA1-IgG ICs, but not Gd-IgA1 alone, induced renal injury with the development of mesangial immunodeposits of IgA and IgG with C3. Therefore formation of Gd-IgA1-IgG ICs might play a crucial role in the pathogenesis of IgAN [10]. Furthermore, injection of Gd-IgA1-IgG ICs could activate complement, as evidenced by C3 codeposits. Previous studies have also indicated that IgA-containing ICs could activate complement through the alternative pathway and the lectin pathway [17–19]. However, the mechanism of complement activation in the pathogenesis of IgAN has not been determined. Future studies are required to clarify the relationship between Gd-IgA1-IgG ICs and complement activation.

Glomerular endothelial cells are a significant contributor to the glomerular filtration barrier. The glomerulus is a structure in which the capillary wall acts as an efficient filtration barrier to restrict the passage of larger molecules and primarily proteins, but it is more permeable to water and small molecules [20]. Glomerular endothelial cells, glomerular basement membrane and podocytes, with their interdigitated foot processes, contribute to these functions. However, it should be noted that deposits from blood vessels in the mesangial region are mediated only by endothelial cells. Therefore we focused on endothelial cell injury to clarify the deposition of Igs in the mesangial region.

Endothelial glycocalyx is mainly composed of proteoglycans and glycoproteins that cover the cell surface of endothelial cells in the capillaries. The glycocalyx modulates leukocyte adhesion and migration and thrombus formation and functions as a vascular barrier [21]. Glycocalyx loss is associated with increased vascular permeability and contributes to the development of renal injuries. Studies of aging Munich Wistar Frömter rats, a model of spontaneous albuminuric chronic kidney disease, revealed that extensive loss of endothelial glycocalyx correlates with defects in microvascular permeability to albumin [15]. Diabetic patients show damage to endothelial glycocalyx, the severity of which correlates with the presence of microalbuminuria [22]. Therefore we assessed glycocalyx loss by real-time imaging to evaluate endothelial cell injury mediated by Gd-IgA1-IgG IC deposition. This imaging study showed that Gd-IgA1-IgG ICs, but not Gd-IgA1 alone, rapidly induced removal of the surface layer, glycocalyx, of renal microvascular endothelium. Furthermore, the glycocalyx loss was maintained for some time, after which Gd-IgA1-IgG ICs deposited in the mesangial region. The deposition of Gd-IgA1-IgG ICs was consistent with a previous report that identified IgG specific for Gd-IgA1 in all IgAN-biopsy specimens [23]. These results suggest that Gd-IgA1-IgG ICs disrupt the function of glomerular endothelial cells and alter vascular permeability, which then trigger IgA deposition in the mesangial region.

It has been demonstrated that injection of nephritogenic IgA from IgAN-prone mice induces endothelial cell activation and causes immediate deposition of nephritogenic IgA along the glomerular capillary wall [9]. Nephritogenic IgA might have an affinity for glomerular endothelial cells to develop glomerular injury. This study suggests that human Gd-IgA1-IgG ICs also activate glomerular endothelial cells. In fact, Gd-IgA1-IgG ICs elevated transcription of several genes encoding endothelial adhesion molecules and proinflammatory cytokines.

In conclusion, Gd-IgA1-containing ICs, but not free Gd-IgA1, activated glomerular endothelial cells of nude mice and induced elevated expression of proinflammatory cytokines, chemokines and adhesion molecules. These Gd-IgA1-containing ICs thus induced endothelial injury that altered the permeability of endothelium and favor entry of ICs in the mesangial region. These findings may aid the development of new therapeutic approaches to protect glomerular endothelial cells from injury in IgAN.
